# Isolation and enrichment of melanocytes from human corneal limbus using CD117 (c-Kit) as selection marker

**DOI:** 10.1038/s41598-020-74869-1

**Published:** 2020-10-16

**Authors:** Naresh Polisetti, Ursula Schlötzer-Schrehardt, Thomas Reinhard, Günther Schlunck

**Affiliations:** 1 Eye Center, Medical Center, Faculty of Medicine, University of Freiburg, Killianstrasse 5, 79106 Freiburg, Germany; 2 Department of Ophthalmology, University Hospital Erlangen, Friedrich-Alexander-University of Erlangen-Nürnberg, Schwabachanlage 6, 91054 Erlangen, Germany

**Keywords:** Cell growth, Biomaterials, Biomaterials - cells, Tissue engineering, Regenerative medicine, Biotechnology, Cell biology, Immunohistochemistry, Biological techniques, Immunological techniques, Corneal diseases, Eye diseases

## Abstract

Limbal melanocytes (LM) are located in the basal epithelial layer of the corneoscleral limbus and interact with adjacent limbal epithelial progenitor cells. The exploration of their biological role in the maintenance of the limbal stem cell niche has been limited by the difficulty of LM isolation and cultivation. Here, we report on a facile protocol for the efficient isolation and enrichment of pure populations of human LMs by fluorescence-activated cell sorting (FACS) using antibodies raised against the cell surface marker CD117 (c-Kit). The enriched LMs retain self-renewal capacity and sustained melanin production, and are suitable to study the potential of LMs in stem cell-based corneal tissue engineering.

## Introduction

Limbal melanocytes (LM) are embedded in the basal limbal epithelium and interact with adjacent limbal epithelial progenitor cells (LEPC). LM-derived melanin granules, transferred to LEPC, were suggested to serve as scavengers of reactive oxygen to prevent DNA damage^1,2^. LMs also migrate towards the central cornea after significant epithelial disruption^3,4^. Besides their protective effects, LMs also play an important role in maintaining the stemness state of LEPC both in vitro and in vivo^5,6^. Hayashi and coworkers reported that N-cadherin-positive LMs co-localized with CK15/N-cadherin-positive limbal basal epithelial cells and that N-cadherin-mediated cell–cell interactions are important in maintaining the potency of the epithelial stem cells^7^. LMs are enriched in limbal crypts which also harbor the most pigmented and immature LEPCs^5,8^. LMs from highly pigmented limbal biopsies were shown to support the stemness state and growth of LEPC and to promote the formation of multi-layered epithelial cell sheets^5^. The cultivation of melanocytes has proven to be difficult due to the low percentage of melanocytes in the total epithelial cell population and their low mitotic activity compared with the other cell types^9^. This typically results in a rapid overgrowth of melanocyte cultures by keratinocytes and fibroblasts^10^.

Various methods have been suggested to obtain pure melanocyte cultures from epidermal and limbal epithelia, the most common ones rely on differential cytotoxic effects of G418 (geneticin) to prevent rapid overgrowth by epithelial cells and fibroblasts^5,11–14^. Although most of these cytotoxic procedures provide pure melanocyte populations, subtle toxic side effects on the phenotype and functional properties of the surviving cells remain a possibility^14^. To avoid these limitations, Hayashi and coworkers used fluorescence-activated cell sorting (FACS) for isolation of LMs based on N-cadherin expression^7^. However, this approach is limited by the fact that only a small fraction of LMs expresses N-cadherin^7^. Recently, Willemsen and coworkers have reported the instant isolation of highly purified human melanocytes from freshly prepared epidermal cell suspensions by means of FACS using CD117 as a selection marker^14^. CD117 (c-Kit) is expressed on both melanocytes and several stem cell populations, and binding to its ligand stem cell factor (SCF) plays an important role in cell homeostasis, including in melanocytes^15,16^. CD117 expression was also observed in LMs, and CD117/SCF signaling was shown to play an important role in the limbal stem cell niche^5,17^.

The aim of this study was to establish and validate a facile technique for the rapid isolation of pure human LM populations from organ-cultured corneal samples by means of FACS using the CD117 selection marker. CD117^+^ cells were expanded on recombinant E8 fragments of laminin-511 (LN-511-E8), a basement membrane (BM) component of various stem cell niches including the limbal niche^18^. The sorted CD117^+^ cells were characterized by the expression of established melanocyte markers at the cellular and molecular level. The cell growth characteristics and functional properties of LMs also analyzed in this study. This protocol resulted in obtaining a pure population of functional LMs in a very short period of time and can be used for functional studies to further examine the role of melanocytes in the limbal stem cell niche.

## Results

### Localization of melanocytes and CD117 expression in situ

**Figure 1:**
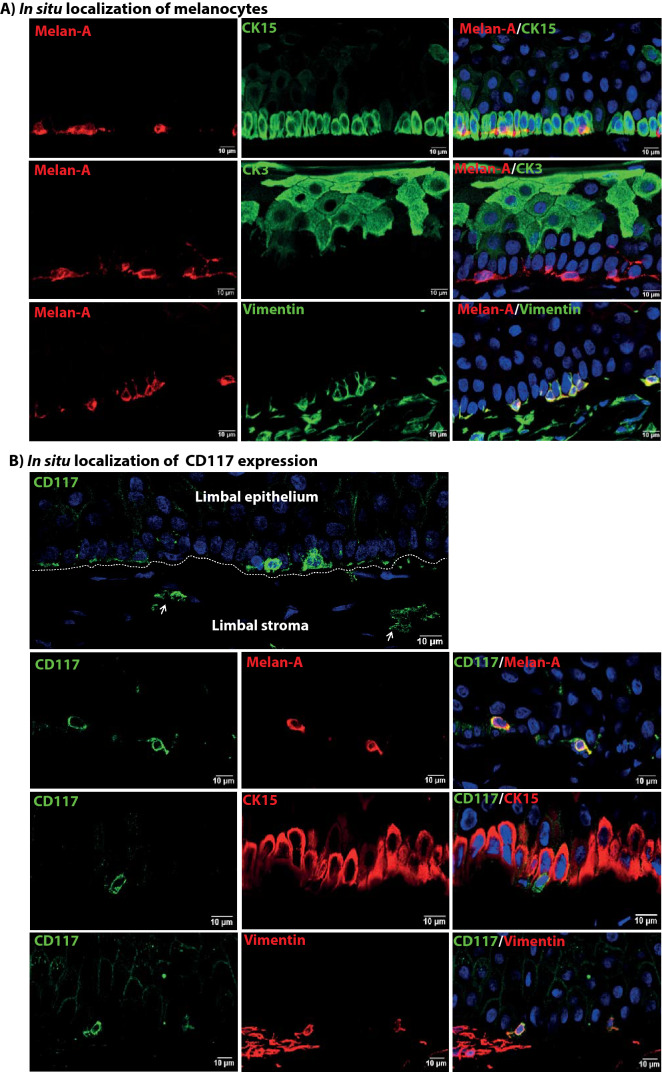
Localization of melanocytes and CD117 expression in situ*.* (**A**) Immunohistochemical analysis of corneoscleral tissue sections showing melanocytes (red) colocalize with clusters of tightly packed cytokeratin(CK)15^+^ (green) limbal epithelial progenitor cells (z-stack image) but not with CK3^+^ (green) cells. Melanocytes in the basal layers (red) colocalize with vimentin (green), but subepithelial vimentin^+^ stromal cells not showing expression of Melan-A (z-stack image). Nuclear counterstaining with 4′,6‐diamidino‐2‐phenylindole (DAPI, blue). (**B**) Immunohistochemistry of tissue sections showing the presence of CD117^+^ cells (green) in the basal layer of limbal epithelium and in the blood vessels of limbal stroma (white arrows) (z-stack image). The dotted line represents the basement membrane. Immunofluorescence double labeling of corneoscleral tissue sections showing coexpression of CD117^+^ cells (green) with Melan-A (red), vimentin (red) but not with epithelial progenitor marker CK15 (red). CD117 expression not observed in the subepithelial vimentin^+^ stromal cells (red). Nuclei are counterstained with DAPI (blue).

Immunohistochemical stains of corneoscleral tissue sections revealed that melanocytes (Melan-A^+^) occurred throughout the basal limbal epithelial layer (cytokeratin (CK)-15^+^) but were more frequent in crypts as compared to non-crypt regions. Melanocytes (Melan-A^+^, red) colocalize with clusters of tightly packed CK15^+^ LEPC cells (green) at the edge of limbal crypts and not with more superficial CK3^+^ cells (green) (Fig. 1A). Melanocytes in the basal layer coexpress Melan-A and vimentin (green), whereas Melan-A is absent in subepithelial vimentin^+^ cells in the limbal stroma (Fig. 1A).

CD117 was expressed in cells interspersed within the basal layer of the limbal epithelium (green) as well as in blood vessels of the limbal stroma (white arrows) (Fig. 1B). Double immunostaining confirmed colocalization of the melanocyte marker Melan-A (red) and CD117 (green) (Fig. 1B). Similarly, cells coexpressing vimentin and CD117 were found in the basal epithelial layer. The epithelial progenitor marker CK15 (red) did not colocalize with CD117, but CK15^+^ and CD117^+^ cells were in close contact (Fig. 1B). Subepithelial vimentin^+^ cells did not show expression of CD117 (Fig. 1B).

### Flow cytometry analysis and sorting of CD117^+^ cells

**Figure 2:**
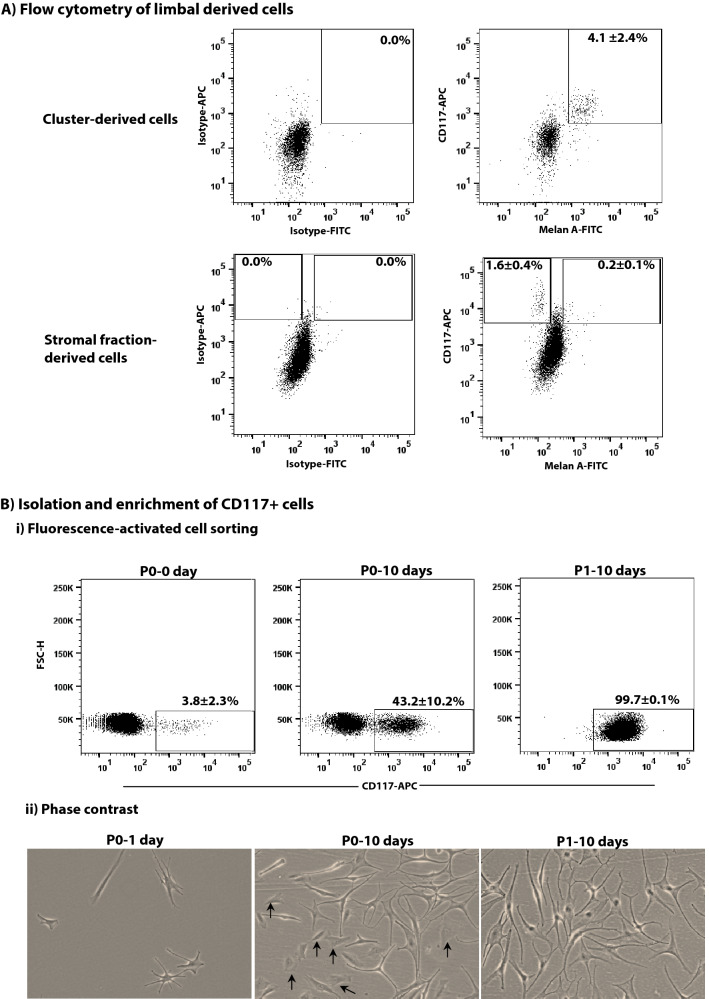
Flow cytometry of limbal cells and sorting. (**A**) Flow cytometry analyses of cluster derived limbal cells showing a high percentage of CD117^+^/Melan-A^+^ cells compared to stromal fraction-derived cells. Stromal fraction-derived cells also showing the presence of CD117^+^/Melan-A^−^ cells. Percentages (%) of positive cells are expressed as the means ± SEM (n = 4). (**B**) Fluorescent cell sorting of cluster derived cells (i, P0–0 day), enrichment of CD117^+^ cells showing a gradual increase (i, P0–10 days), finally yielded a pure population of CD117^+^ cells (i, P1–10 days). Percentages (%) of positive cells are expressed as the means ± SEM percentage (%) (n = 7) (i). Phase-contrast images showing the attached multidendritic cells after 24 h of seeding of CD117^+^ cells (ii, P0–1 day) and stromal contamination after a few days in culture (black arrows) (ii, P0–10 days). Sorting of cultured cells showing the absence of fibroblast and all cells showing large bodies, flattened, smooth, multiple dendritic morphologies (ii, P1–10 days). Magnification × 100.

Limbal cluster-derived and stromal fraction-derived single cells were stained for CD117 and Melan-A. Subsequent flow cytometry analysis revealed that 4.1 ± 2.4% of limbal cluster-derived cells and 0.2 ± 0.1% of stromal fraction-derived cells expressed CD117^+^/Melan-A^+^ (n = 4) (Fig. 2A). Interestingly, we observed CD117^+^/Melan-A^-^ staining in 1.6 ± 0.4% of the cells in the stromal fraction probably representing vascular endothelial cells described above (Fig. 2A). Based on these observations, we used the CD117^+^/Melan-A^+^ fraction of limbal cluster-derived cells for the efficient isolation of LMs.

Limbal cluster-derived cell suspensions from donor corneal samples provided a yield of 1.0 to 7.0% (3.8% ± 2.3%) of CD117^+^ cells (Fig. 2B-i, P0–0 day; n = 7). The CD117^+^ cells were seeded on LN-511-E8 coated culture plates in CNT-40 medium. Spreading of CD117^+^ cells occurred two hours after plating and the cultures consisted of cells with large, flattened, smooth bodies with multiple dendrites, the characteristic feature of melanocytes, after 24 h (Fig. 2B-ii, P0–1 day). After a few days of culture, we observed stromal/fibroblast-like cell growth along with melanocyte growth in most of the cultures (6/7) (Fig. 2B-ii, P0–10 days). To remove contaminating fibroblastoid cells, we performed a second FACS sorting procedure. The isolated CD117^+^ cells (43.2 ± 10.2%; Fig. 2B-i, P0–10 days) cultured on LN-511-E8 for 10 days contained 99.7 ± 0.1% CD117^+^ cells (Fig. 2B-i, P1–10 days). The phase-contrast image also confirmed the absence of contaminating cells (Fig. 2B-ii, P1–10 days).

### Characterization of CD117^+^ enriched cell populations

**Figure 3:**
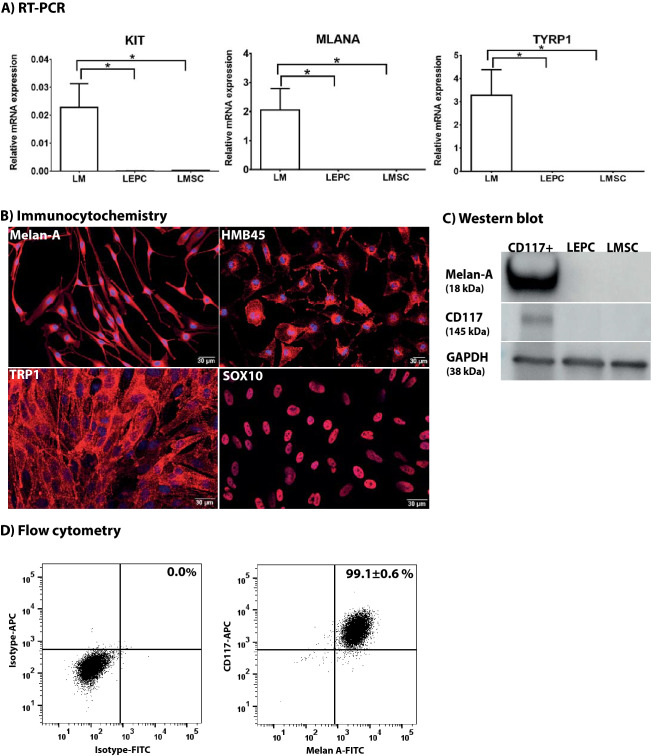
Characterization of cultured CD117^+^ cells for melanocyte characteristics. (**A**) Quantitative real-time polymerase chain reaction (qRT-PCR) primer assays confirm the differential expression of established *melanocyte markers *(*KIT, MLANA, TYRP1*)* in cultured LEPC*, LMSC, and LMs. Data are expressed as means (2^−ΔCT^) ± SEM (*n* = 5). *p < 0.05; Mann–Whitney *U* test. (**B**) Immunocytochemical analysis of cultured CD117^+^ cells showing expression of melanocyte markers Melan-A, Sox-10, TRP1, and HMB45 (red); nuclear counterstaining with 4′,6‐diamidino‐2‐phenylindole (blue) (**C**) CD117 and Melan-A protein expression in CD117^+^ cells were confirmed by western blot analysis. Reprobing with an anti-GAPDH antibody served as a control. Uncropped versions of Western blot are shown in Supplementary Figure 1. (**D**) Flow cytometry analysis for double staining for CD117 and Melan-A markers in cells expanded. Data are expressed as a percentage as means ± SEM. LEPC, limbal epithelial progenitor cells; LMSC, limbal mesenchymal stromal cells; LM, limbal melanocytes; KIT, CD117; MLANA, Melan-A; SOX10, sex-related HMG box 10; TYRP1 or TRP1, tyrosinase-related protein 1; HMB45, human melanoma black-45; GAPDH; Glyceraldehyde 3-phosphate dehydrogenase).

To verify the phenotype of CD117^+^ enriched putative LM cell populations, established melanocyte markers were studied by gene expression analysis, flow cytometry, immunocytochemistry, and Western blotting. Gene expression analysis comparing CD117^+^ enriched populations with LEPC and LMSC populations by quantitative real-time polymerase chain reaction (qRT-PCR) showed significantly higher expression levels (> 100-fold) of melanocyte markers CD117/c-Kit (KIT), Melan-A (MLANA) and tyrosine-related protein (TYRP1) in putative LMs compared to LMSCs and LEPCs (Fig. 3A). For immunostaining, LMs were cultured on four-well chamber slides in the presence or absence of LN-511-E8 as a substrate. Immunostaining confirmed the expression of Melan-A (P3), SRY-box transcription factor 10 (SOX10), human melanoma black-45 (HMB45) (P2, LN-511-E8), and tyrosinase-related protein 1 (TRP1, P3, plated at high-density) in all cultured CD117^+^ cells suggesting pure melanocyte cultures (Fig. 3B). Western blotting confirmed the presence of CD117 and Melan-A exclusively in CD117^+^ enriched cell populations (Fig. 3C). Flow cytometry analysis of the CD117^+^ enriched cell cultures double-stained for CD117 and Melan-A indicated that 99.1 ± 0.6% of all cells expressed both CD117 and Melan-A (Fig. 3D).

### Growth potential and melanin synthesis of CD117^+^ cell populations

**Figure 4:**
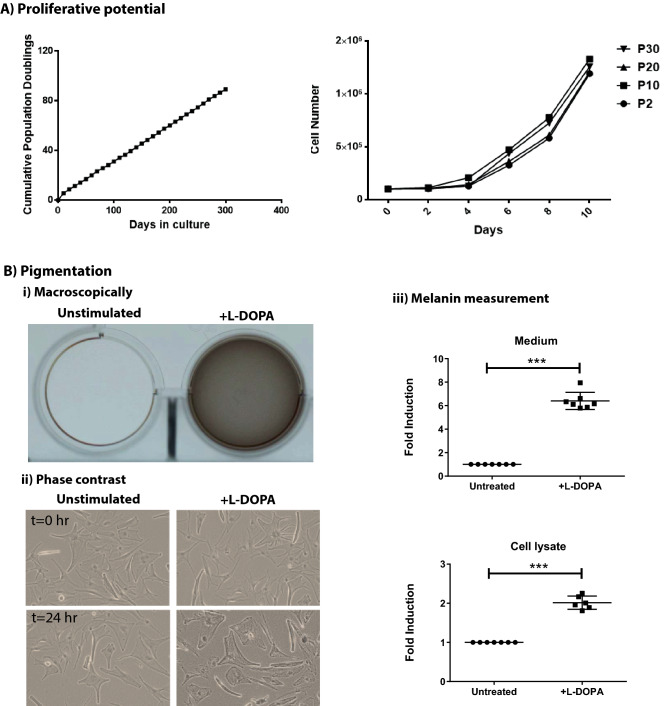
Culture characteristics of melanocytes and melanin production. (**A**) The cumulative population doubling versus duration of culture demonstrates a relatively linear increasing population doubling rate with the progression of time (left). Limbal melanocytes (LM) from passages 2 (P2), P10, P20, and P30 showing similar proliferative potential (right). Data are shown as mean ± SEM (n = 5). (**B**) LMs were cultured in the presence or absence of 1 mM L-DOPA for 24 h. l-DOPA treated cells produced melanin, causing light brown coloring of the culture medium, as can be observed macroscopically (i). Phase-contrast images also show the darkening of the cells 24 h after treatment with l-DOPA compared with 0 h (ii). l-DOPA stimulation significantly increased the intracellular melanin concentration (twofold), whereas the melanin concentration in the medium was increased to sixfold compared to unstimulated condition (iii). Data are expressed as means ± SEM (n = 6) *p < 0.05; **p < 0.005; Mann–Whitney *U* test.

Pure LM cultures could be passaged for 30 generations with 100 population doublings over 10 months and doubling times of 2.78 ± 0.5 days (Fig. 4A, left column). Moreover, LMs showed similar proliferation potential from passage 2 to passage 30 (Fig. 4A, right column), although they exhibited signs of growth arrest by contact inhibition. We also observed that the LMs did not show any evident changes in morphology between passages.

L-DOPA stimulated melanin production by cultivated LMs (P2 to P30), which was indicated by macroscopic darkening of the culture medium (Fig. 4B-i) and by microscopic appearance of intracellular granules after stimulation (Fig. 4B-ii). Whereas the cellular melanin (cell lysate) content was increased twofold in L-DOPA treated cells compared to untreated cells (Fig. 4B-iii), a sixfold increase was observed in conditioned medium of treated cells compared to control medium (Fig. 4B-iii). These data show that enriched CD117^+^ LMs are functional in producing and secreting melanin into the culture medium.

## Discussion

The limbal stem cell niche is a complex microenvironment composed of specific extracellular matrix (ECM) components and limbal niche cells comprising LEPC, intraepithelial LMs, subepithelial LMSCs and transmigrating immune cells^19–23^. Human LEPCs and LMSCs were successfully grown in culture^18,24^, but LMs have proven difficult to culture efficiently^5^. This may be due to their low numbers in the tissue and a low proliferation rate leading to frequent overgrowth by epithelial cells or fibroblasts in vitro^5,7^. Robust and reliable methods to propagate LMs in vitro would foster a better understanding of their functional roles in limbal stem cell niche maintenance and disease states. Differential trypsinization with low concentrations of trypsin has been suggested to release melanocyte-like cells and stromal fibroblast-like cells for further cultivation, leaving contaminating epithelial cells behind^25^. However, due to their high proliferation rate, contaminating fibroblasts are the main obstacle to achieve pure melanocyte cultures. Several approaches were used to remove contaminating fibroblasts, most commonly the addition of geneticin to inhibit protein synthesis^5,26–28^. At low concentration, geneticin has very limited toxicity for melanocytes showing lower protein synthesis but causes harm to actively synthesizing fibroblast and stromal cells^26,29^. Although this procedure provides pure melanocyte populations, it is very time-consuming and may also affect the phenotype and functional properties of the melanocytes, since the action of geneticin is not cell type-specific^14,28^.

In this study, we established and validated a novel, alternative method for the isolation and efficient enrichment of melanocytes from the corneal limbus. We used the enzyme collagenase A to isolate limbal cell clusters. Earlier studies have shown that clusters derived by collagenase digestion included more epithelial progenitor cells, LMSCs, and melanocytes than dispase-isolated cell sheets^23,30^. We then targeted the antigen CD117, a cell surface marker expressed in many stem and progenitor cells including melanocytes^5,14^, to isolate LMs from limbal tissue using FACS sorting. Collagenase digestion of limbal tissue results in single-cell suspensions of the limbal stroma (stromal fraction-derived) and limbal epithelial niche cell clusters containing LEPC, LMSC, and LM^18,23^. We observed that cluster-derived cell suspensions contained more CD117^+^/Melan-A^+^ cells than the stromal fraction. Hence, we used the cluster-derived cells for FACS sorting to efficiently isolate LMs. The fraction of CD117^+^ cells in cluster-derived cells varied from 1 to 7.0%, most likely due to donor age, tissue quality, and duration of organ culture. The cultured cell populations still contained small amounts of contaminating stromal cells in most of the cultures (6/7 cultures). Therefore, a second CD117-based FACS sort was performed after 10 days to yield a pure population of LMs as confirmed by the expression of melanocytic markers upon qRT-PCR, immunocytochemistry, flow cytometry, and Western blotting^5^.

Since ECM regulates cell survival, proliferation, and differentiation^31,32^, niche-specific ECM substrates are needed to promote cell propagation in vitro. Laminins are the best described ECM constituents present in BM of adult stem cell niches, including the limbal stem cell niche^18,21^. We reported earlier that the LN-α5 chain is preferentially localized to the BM of the limbal epithelium compared to the corneal epithelium^18^. We have also reported that LN-α5 containing isoform, LN-511, and their biologically active E8 fragments, support the efficient expansion of LEPC^18^. As LMs also reside on the epithelial BM in close association with LEPC in vivo, we used LN-511-E8 for LM expansion in vitro. Our data clearly suggest that LN-511-E8 supports the long term expansion of LMs. Typically, the growth of melanocytes is slow and related to the age of the donor. Neonatal dermal melanocytes can undergo at least 50 population doublings during a period of 6 months, whereas dermal melanocytes from adult skin were reported to proliferate for only 1 month^33^. Uveal melanocytes from adult sources had a doubling time of 3–4 days and were passaged for 23 generations with 35 population doublings over 7 months^34^. In our current study, LMs from cadaveric corneas (average donor age 72.3 years) had a population doubling time as short as 2–3 days, and could be passaged for 30 generations with 100 population doublings over 10 months with unabated proliferative potential. These findings demonstrate that our isolation protocol and subsequent expansion on LN-511-E8 coated plates support long term survival and proliferation of LMs. On the other hand, we also observed signs of growth arrest by contact inhibition, which is a powerful anticancer mechanism that is lost in cancer cells^35^. Moreover, we did not notice any increased cell proliferation in long term LM cultures, which would be one of the associated factors of genomic instability and tumor growth^36^. These observations argue against significant genomic instability in long term LM cultures. A main function of melanocytes is melanin production, which plays a crucial role in many biological processes especially protection against the detrimental effects of UV radiation. In epidermal melanocytes, L-DOPA treatment was reported to induce a 4-fold increase in cellular melanin and a 26-fold increase in secreted melanin^14^. In line with these observations, CD117^+^ LMs treated with L-DOPA showed a twofold increase in cellular melanin and a sixfold increase in melanin released into the culture medium. These data show that isolated CD117^+^ LMs are functional in producing and secreting melanin. The protocol has the advantage of rendering pure melanocyte populations within 10 days as compared to 2–3 months needed for a sequence of geneticin treatments^37^.

In conclusion, we propose a new protocol for the isolation and propagation of highly purified populations of primary human LMs by taking advantage of their cell surface expression of CD117 and recombinant LN-511-E8 as a culture substrate. With our protocol, it is possible to obtain large numbers of purified LMs from organ cultured corneal scleral rims. Cells obtained this way expressed established melanocytic markers, showed a multi-dendritic phenotype, sustained self-renewal potential, and upregulated melanin production upon stimulation. The protocol presented carries the advantage to obtain LMs in a very short period of time and to avoid any toxic effects of commonly used selection agents. The pure LM populations can be used for stem cell-based tissue engineering approaches and functional studies to further examine the role of melanocytes in the limbal stem cell niche.

## Materials and methods

Human donor cornea tissue with appropriate research consent was provided by the Lions Cornea Bank Baden-Württemberg after retrieval of corneal endothelial transplants. Informed consent to corneal tissue donation had been obtained from the donors or their relatives. Experiments using human tissue samples were approved by the Institutional Review Board of the Medical Faculty of the University of Freiburg (25/20) and adhered to the tenets of the Declaration of Helsinki. No organs/tissues were procured from prisoners.

### Cell isolation

Limbal cells were isolated as previously described^18^. Briefly, organ-cultured corneoscleral tissue (Table 1; mean age 72.7 ± 10.45 years; culture duration 27.2 ± 7.5 days; light pigmented donor limbal tissue) was cut into 12 three-clock-hour sectors, from which limbal segments were obtained by incisions made at 1 mm before and beyond the anatomical limbus. Limbal segments were enzymatically digested with collagenase A (Sigma-Aldrich; 2 mg/ml) at 37 °C for 18 h to generate clusters containing mixtures of epithelial, mesenchymal and melanocytic cells. Cell clusters were separated from single cells (referred to as stromal fraction-derived cells) by using reversible cell strainers with a pore size of 37 µm (Stem Cell Technologies, Köln, Germany). Subsequently, the cell clusters were dissociated into single cells (referred to as cluster-derived cells) by digestion with 0.25% trypsin and 0.02% EDTA (Pan Biotech, Aidenbach, Germany) at 37 °C for 10–15 min.

**Table 1:** Organ cultured corneal scleral tissues used in this study.

S. no.	Age (years)	Post mortem time (h)	Duration of cultivation (days)
1	47.7	24.5	30.5
2	47.7	26.5	30.5
3	69.7	29.5	27.3
4	78.5	24.6	25
5	97.8	29.5	81.3
6	84.8	29.6	27.3
7	79.6	29.5	26.4
8	79.6	31.5	22.5
9	61.0	26.4	21.5
10	79.6	27.4	23.5
11	61.0	21.5	22.5
12	81.2	25.5	22.4
13	58.6	30.5	29.5
14	65.0	27.3	63.5
15	62.3	25	30.5
16	76.0	0	23.5
17	78.8	75.3	28.4
18	65.2	0	27.5
19	76.7	27.3	25.3
20	74.6	27.3	24.6
21	65.2	22.5	29.5
22	76.7	21.5	27.3
23	80.5	0	21.3
24	74.8	83.4	22.5
25	74.8	83.4	22.5
26	77.0	29.5	33.4
27	75.5	30.5	22.5
28	79.2	23.5	21.6
29	75.8	25.3	27.4
30	75.8	24.6	27.4

To isolate LMs from the cluster-derived single-cell suspension, we used fluorescence-activated cell sorting (FACS) to enrich CD117^+^ cells (see below). After sorting, CD117^+^ cells were seeded in LN-511-E8 (iMatrix-511, Nippo; 0.5 µg/cm^2^) coated T75 flasks (Corning, Tewksbury, MA) and cultured in CNT-40 medium (CellnTec, Switzerland) at 37 °C, 5% CO_2_ and 95% humidity. The medium was changed every other day. For further enrichment, the cultures underwent the second cycle of FACS sorting for CD117^+^ cells after 10 days of cultivation and subsequent expansion in the aforementioned conditions on LN-511-E8. All the following expeirments on LMs carried out on LN-511-E8 substrate, except as noted.

To enrich LEPC and the associated mesenchymal stromal cells (LMSC), cluster-derived single-cell suspensions were seeded into T75 flasks in Keratinocyte serum-free medium (KSFM) supplemented with bovine pituitary extract, epidermal growth factor (Life Technologies), or Mesencult media (Stem Cell Technologies), respectively. The cultures were incubated at 37 °C, 5% CO_2_, and 95% humidity. The media was changed every other day.

### Fluorescence-activated cell sorting (FACS)

Single-cell suspensions were incubated with FcR blocking reagent (Miltenyi Biotec, Bergisch Gladbach, Germany; 20 μl/10^6^ cells) for 5 min. Subsequently, cells were washed and incubated with a mouse anti-human CD117-APC antibody (10 μl/10^6^ cells) (Miltenyi Biotec) in 100 µl phosphate-buffered saline (PBS), 0.1% sodium azide and 2% fetal calf serum for 30 min at 4 °C in the dark. Cells were then washed and FACS was performed using a FACS Aria II sorter (BD Biosciences, Heidelberg, Germany) and the FACSDiva software (BD Pharmingen; BD Biosciences). Post-acquisition analysis was performed using FlowJo software (Tree Star, Inc., Ashland, OR).

### Flow cytometry

Cells were characterized by flow cytometry using fluorochrome-labeled antibodies and isotype control antibodies (BD Biosciences). Single-cell suspensions (0.5–1 × 10^6^ cells) were incubated with CD117-APC as mentioned above. After three washes, melanin staining was carried out using the BD Fixation/Permeabilization solution kit (BD Biosciences) as per the manufacturer’s instructions. Briefly, cells were permeabilized using the Fixation/Permeabilization buffer and stained with an anti-Melan-A-FITC antibody (Novus Biologicals, Wiesbaden Nordenstadt, Germany) for 30 min. After three washes, cells were resuspended in ice-cold PBS, and flow cytometry was performed on a FACSCanto II (BD Biosciences) by using FACS Diva Software (BD FACSDiva 8.0.1; https://www.bdbiosciences.com/en-us/instruments/research-instruments/research-software/flow-cytometry-acquisition/facsdiva-software). A total of 10,000 events were acquired. Post-acquisition analysis was performed using FlowJo software (FlowJo 10.2, Tree Star Inc. https://www.flowjo.com/).

### Real-time RT-PCR

RNA was isolated from cultured cells using the RNeasy Micro Kit (Qiagen, Hilden, Germany) including an on-column DNase digestion step according to the manufacturer’s instructions. First-strand cDNA synthesis was performed using 2 µg of RNA and Superscript II reverse transcriptase (Invitrogen, Karlsruhe, Germany) as previously described^23^. PCR reactions were run in triplicate using TaqMan Probe Mastermix (Roche Diagnostics, Mannheim, Germany), according to the manufacturers’ recommendations. Primer sequences (Sigma-Aldrich) are given in Table 2. For normalization of gene expression levels, ratios relative to the housekeeping gene *GAPDH* were calculated by the comparative *C*_T_ method (ΔΔ*C*_T_). Genes were considered as differentially expressed when their expression levels exceeded a two-fold difference in all specimens analyzed (n = 5).

**Table 2:** Primers used in qRT-PCR primer assays.

Gene symbol	Accession no.	Product length	Probe no.	Sequence 5′–3′
GAPDH	NM_002046.3	72	P3	CAGCAAGAGCACAAGAGGAA
				GTGGTGGGGGACTGAGTGT
KIT	NM_000222.2	61	P6	GAGTAGCTTACCAGAAGCTTCCATAG
				CATAGGGACTGATGCCTTCC
MLANA	NM_005511.1	79	P39	GAGAAAAACTGTGAACCTGTGGT
				AAGGTGGTGGTGACTGTTCTG
TYRP1	NM_000550.2	77	P2	CCTGTGACCAGAGGGTTCTC
				CCGGACAAAGTGGTTCTTTTC

Primers were used for Probe based (Universal Probe Library) qRT-PCR assays with an annealing temperature of 60 °C.

### Western blot

Total protein was isolated from cells using RIPA buffer (R0278, Sigma-Aldrich) containing a protease inhibitor cocktail (complete Tablets Mini, Roche, Basel, Switzerland). The total protein concentration was evaluated in a colorimetric assay (PierceTM BCA Protein Assay Kit, Thermo Fisher Scientific). Ten micrograms of total protein were separated by SDS-PAGE under reducing conditions, and immunoblot analyses were performed using antibodies (Table 3) against CD117 (1:1000), Melan-A (1:10,000), and GAPDH (1:50,000) followed by horseradish peroxidase-labeled anti-mouse or rabbit IgG (Jackson ImmunoResearch). Protein bands were visualized using the enhanced chemiluminescence Western blot detection reagent (GE Healthcare) and the fusion fx Imager/fusion software (Fusion FX7 Edge 18.05, Vilber Lourmat; https://www.vilber.com/fusion-fx/).

**Table 3:** List of antibodies used.

Antibody (clone), host species	Antibody concentration	Application	Antibody source
CD117 (c-Kit), APC (A3C6E2), mouse	10 µl/10^6^ cells	Flow cytometry	Miltenyi Biotec
IgG3 isotype FITC (MG3-35), mouse	5 µg/ml	Flow cytometry	Biolegend
IgG1 isotype APC (MOPC-21), mouse	20 µl/10^6^ cells	Flow cytometry	BD
Melan-A FITC, (MLANA/788), mouse	5 µg/ml	Flow cytometry	Novus Biologicals
Cytokeratin 3 (AE5), mouse	1:100	Immunohistochemistry	Abcam
Cytokeratin 15 (LHK15), mouse	1:500	Immunohistochemistry	Abcam
Cytokeratin 15 (EPR1614Y), rabbit	1:500	Immunohistochemistry	Abcam
Vimentin, (D21H3), rabbit	1:500	Immunohistochemistry	Cell Signaling
HMB-45 (HMB45), mouse	0.5 µg/ml	Immunocytochemistry	Abcam
Sox10 (EPR4007), rabbit	1:250	Immunocytochemistry	Abcam
TRP1 (EPR21960), rabbit	1:1000	Immunocytochemistry	Abcam
CD117/c-kit (K45), mouse	1:100	Immunohisto/cytochemistry	Invitrogen
Mouse IgG1k	100 µg/ml	Immunohisto/cytochemistry	BD
Melan-A (OTI3E2), mouse	1:100	Immunohisto/cytochemistry	ORIGENE
GAPDH; (6C5), mouse	1:50,000	Western blot	Millipore
CD117/c-Kit (Ab81), mouse	1:500 1.1000	Immunohisto/cytochemistry, Western blotting	Cell Signaling
Melan A, (EPR20380), rabbit	1:1000 1:10,000	Immunohisto/cytochemistry, Western blotting	Abcam

### Immunohisto- and immunocytochemistry

Corneoscleral tissue samples (mean age 75.2 ± 10.9 years) within 16 h after death were embedded in optimal cutting temperature (OCT) compound and frozen in liquid nitrogen. Cryosections of 6 μm thickness were cut from the superior or inferior quadrants and LMs cultured on 4 well-glass chamber slides (LabTek; Nunc, Wiesbaden, Germany) were fixed in 4% paraformaldehyde for 15 min, blocked with 10% normal goat serum (NGS), and incubated in primary antibodies (Table 3) diluted in 2% NGS, 0.1% Triton X-100 in PBS overnight at 4 °C or 3 h at room temperature. Antibody binding was detected by Alexa-488- and tetramethylrhodamine (TRITC)-conjugated secondary antibodies (Life Technologies, Carlsbad, CA) and mounted in Vectashield antifade mounting media with DAPI (Vector, Burlingame, CA). Immunolabelled cryosections and cultured LMs were examined with a laser scanning confocal microscope (TCS SP-8, Leica, Wetzlar, Germany). For negative controls, the primary antibodies were replaced by PBS.

### Population doubling assay

A population doubling **(**PD) assay was performed as described previously^24^. Briefly, the assay was performed on LMs from passages 2 to passages 30. Cells were seeded (1 × 10^5^ cells) at each passage and trypsinized after 10 days. The population doubling of cells was calculated as:

$$ {\text{The}}\;{\text{number}}\;{\text{of}}\;{\text{ cell}}\;{\text{doublings}}\;{\text{(NCD)}} = \log_{10} ({\text{y/x}})/\log 10_{2} , $$

where y is the final density of the cells and x is the initial seeding density of the cells. The cumulative population doublings are the sum of PDs in all passages.

Doubling time is calculated from the cell number and the time of cell counting, using the following formula:

$$ {\text{Doubling}}\;{\text{time }} = \, ({\text{t }} - {\text{ t}}_{0} ) \, \log \, 2/(\log \;{\text{y }} - \, \log \;{\text{x}}), $$

where t, t_0_ represents the time at cell counting; y equals the number of cells at time t, and x equals the number of cells at time t_0_.

To determine the proliferative potential of different passages, LMs at Passage 2 (P2), P10, P20, and P30 were seeded (1 × 10^5^) in T75 flasks in triplicates. Cells were trypsinized and the number of cells was determined by days 2, 4, 6, 8, and 10 using a Neubauer counting chamber.

### Melanin production

Melanin production was assessed as described earlier^14^. Briefly, cells were seeded at 1 × 10^5^ cells per well in CNT-40 medium in a 12-well plate and cultured for 24 h at 37 °C in the absence or presence of 1 mM l-3,4-dihydroxyphenylalanine (DOPA) to stimulate melanin synthesis. After 24 h, the culture medium was collected and the adherent cells were washed in PBS. Next, 100 µl of 1 M sodium hydroxide was added to both cells and 100 µl culture medium to dissolve melanin at 70 °C for 90 min. Melanin concentration was determined by comparing 405 nm absorbance values in a Spark microplate reader (TECAN) from experimental samples with a standard curve ranging from 0 to 100 µg/ml generated with synthetic melanin (Sigma). Synthetic melanin was dissolved using 1 M sodium hydroxide solution in water. The fold change values were calculated as OD of the induction/OD of control (n = 7).

### Statistical analysis

The statistical analyses were performed as described earlier^18^. Briefly, the GraphPad InStat statistical package for Windows (Graphpad Software Inc., Version 6.0, La Jolla, CA; https://www.graphpad.com/) was used to perform statistical analyses. Results are expressed as mean ± standard error of the mean (SEM) from individual experiments. The statistical significance (*p* value < 0.05) was determined with the Mann Whitney *U* test.

## Supplementary information

Supplementary Figure 1.

## References

[1] Bessou-Touya S (1998). Chimeric human epidermal reconstructs to study the role of melanocytes and keratinocytes in pigmentation and photoprotection. J. Investig. Dermatol..

[2] Higa K, Shimmura S, Miyashita H, Shimazaki J, Tsubota K (2005). Melanocytes in the corneal limbus interact with K19-positive basal epithelial cells. Exp. Eye Res..

[3] Davies WS, Bailey WH (1954). Malignant melanoma of the cornea; report of a case. AMA Arch. Ophthalmol..

[4] Huang HW, Hu FR, Wang IJ, Hou YC, Chen WL (2010). Migration of limbal melanocytes onto the central cornea after ocular surface reconstruction: an in vivo confocal microscopic case report. Cornea.

[5] Dziasko MA, Tuft SJ, Daniels JT (2015). Limbal melanocytes support limbal epithelial stem cells in 2D and 3D microenvironments. Exp. Eye Res..

[6] Miyashita H (2017). Long-term homeostasis and wound healing in an in vitro epithelial stem cell niche model. Sci. Rep..

[7] Hayashi R (2007). N-Cadherin is expressed by putative stem/progenitor cells and melanocytes in the human limbal epithelial stem cell niche. Stem Cells.

[8] Liu L (2018). Pigmentation is associated with stemness hierarchy of progenitor cells within cultured limbal epithelial cells. Stem Cells.

[9] Tang J, Li Q, Cheng B, Jing L (2014). Primary culture of human face skin melanocytes for the study of hyperpigmentation. Cytotechnology.

[10] Pittelkow MR, Shipley GD (1989). Serum-free culture of normal human melanocytes: growth kinetics and growth factor requirements. J. Cell. Physiol..

[11] Cario M, Taieb A (2019). Isolation and culture of epidermal melanocytes. Methods Mol. Biol..

[12] Godwin LS (2014). Isolation, culture, and transfection of melanocytes. Curr. Protoc. Cell Biol..

[13] Zhang C (2018). A preliminary study of growth characteristics of melanocytes co-cultured with keratinocytes in vitro. J. Cell. Biochem..

[14] Willemsen M, Luiten RM, Teunissen MBM (2020). Instant isolation of highly-purified human melanocytes from freshly-prepared epidermal cell suspensions. Pigment Cell Melanoma Res..

[15] Lennartsson J, Rönnstrand L (2012). Stem cell factor receptor/c-Kit: from basic science to clinical implications. Physiol. Rev..

[16] Grichnik JM, Burch JA, Burchette J, Shea CR (1998). The SCF/KIT pathway plays a critical role in the control of normal human melanocyte homeostasis. J. Investig. Dermatol..

[17] Li G (2018). Human limbal niche cells are a powerful regenerative source for the prevention of limbal stem cell deficiency in a rabbit model. Sci. Rep..

[18] Polisetti N (2017). Laminin-511 and -521-based matrices for efficient ex vivo-expansion of human limbal epithelial progenitor cells. Sci. Rep..

[19] Dua HS, Shanmuganathan VA, Powell-Richard AO, Tighe PJ, Joseph A (2005). Limbal epithelial crypts: a novel anatomical structure and a putative limbal stem cell niche. Br. J. Ophthalmol..

[20] Shortt AJ (2007). Characterization of the limbal epithelial stem cell niche: novel imaging techniques permit in vivo observation and targeted biopsy of limbal epithelial stem cells. Stem Cells.

[21] Schlötzer-Schrehardt U (2007). Characterization of extracellular matrix components in the limbal epithelial stem cell compartment. Exp. Eye Res..

[22] Mei H, Gonzalez S, Deng SX (2012). Extracellular matrix is an important component of limbal stem cell niche. J. Funct. Biomater..

[23] Polisetti N, Zenkel M, Menzel-Severing J, Kruse FE, Schlötzer-Schrehardt U (2016). Cell adhesion molecules and stem cell-niche-interactions in the limbal stem cell niche. Stem Cells.

[24] Polisetty N, Fatima A, Madhira SL, Sangwan VS, Vemuganti GK (2008). Mesenchymal cells from limbal stroma of human eye. Mol. Vis..

[25] Eisinger M, Marko O (1982). Selective proliferation of normal human melanocytes in vitro in the presence of phorbol ester and cholera toxin. Proc. Natl. Acad. Sci. U.S.A..

[26] Halaban R, Alfano FD (1984). Selective elimination of fibroblasts from cultures of normal human melanocytes. In Vitro.

[27] Zhu WY, Zhang RZ, Ma HJ, Wang DG (2004). Isolation and culture of amelanotic melanocytes from human hair follicles. Pigment Cell Res..

[28] Jiarong B, Lei W, Guiwu W, Xueqing L, Fuhe Y (2015). Isolation and culture of melanocytes from the Arctic fox (*Alopex Lagopus*). Ital. J. Anim. Sci..

[29] Horikawa T, Norris DA, Zekman T, Morelli JG (1996). Effective elimination of fibroblasts in cultures of melanocytes by lowering calcium concentration in TPA depleted medium following geneticin treatment. Pigment Cell Res..

[30] Chen SY, Hayashida Y, Chen MY, Xie HT, Tseng SC (2011). A new isolation method of human limbal progenitor cells by maintaining close association with their niche cells. Tissue Eng Part C Methods.

[31] Watt FM, Huck WT (2013). Role of the extracellular matrix in regulating stem cell fate. Nat. Rev. Mol. Cell Biol..

[32] Ahmed M, Ffrench-Constant C (2016). Extracellular matrix regulation of stem cell behavior. Curr. Stem Cell Rep..

[33] Halaban R, Ghosh S, Duray P, Kirkwood JM, Lerner AB (1986). Human melanocytes cultured from nevi and melanomas. J. Investig. Dermatol..

[34] Hu DN, McCormick SA, Ritch R, Pelton-Henrion K (1993). Studies of human uveal melanocytes in vitro: isolation, purification and cultivation of human uveal melanocytes. Investig. Ophthalmol. Vis. Sci..

[35] Abercrombie M (1979). Contact inhibition and malignancy. Nature.

[36] Stopper H, Schmitt E, Gregor C, Mueller SO, Fischer WH (2003). Increased cell proliferation is associated with genomic instability: elevated micronuclei frequencies in estradiol-treated human ovarian cancer cells. Mutagenesis.

[37] Polisetti N, Gießl A, Li S, Sorokin L, Kruse FE, Schlötzer-Schrehardt U (2020). Laminin-511-E8 promotes efficient in vitro expansion of human limbal melanocytes. Sci. Rep..

