# Supplementary material for: Isolation and enrichment of melanocytes from human corneal limbus using CD117 (c-Kit) as selection marker
Source: Sci Rep. 2020 Oct 16;10:17588. doi: 10.1038/s41598-020-74869-1 (PMC7567782; doi:10.1038/s41598-020-74869-1)

Isolation and Enrichment of Melanocytes from Human Corneal Limbus using CD117 (c-Kit) as Selection Marker

Naresh Polisetti, Ursula Schlötzer-Schrehardt, Thomas Reinhard, Günther Schlunck

Supplementary Fig. 1: Uncropped version of Western blot shown in Fig. 3

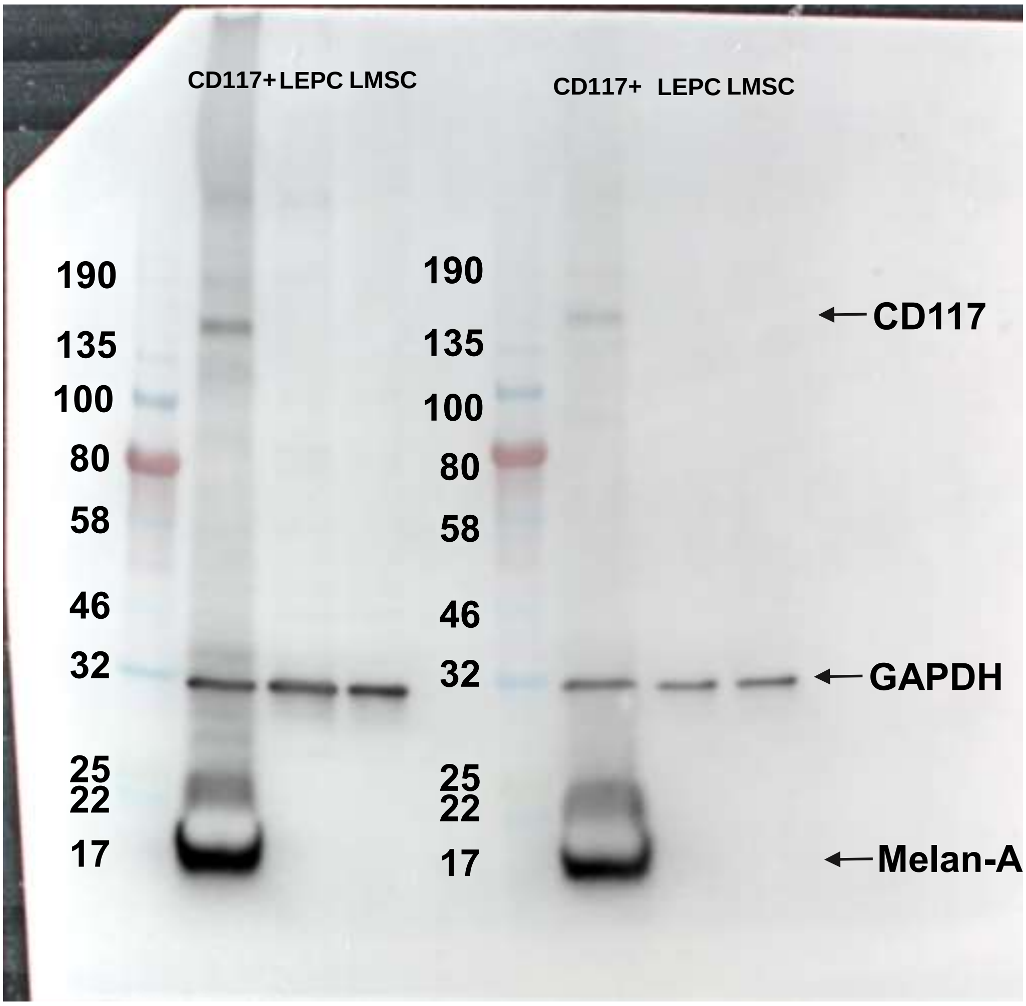

Supplement: Supplementary file 1 — Supplementary Figure 1. [file 41598_2020_74869_MOESM1_ESM.pdf]
